# Increased platelet activation and thrombo-inflammation in early and late-onset preeclampsia

**DOI:** 10.1016/j.rpth.2025.102956

**Published:** 2025-06-24

**Authors:** Kunal Singh, Massimiliano Lia, Akshay Prakasan Sheeja, Martin Federbusch, Anubhuti Gupta, Ahmed Elwakiel, Moritz Köhler, Berend Isermann, Holger Stepan, Shrey Kohli

**Affiliations:** 1 Institute of Laboratory Medicine, Clinical Chemistry, and Molecular Diagnostics, University Hospital Leipzig, Leipzig University, Germany; 2 Leipzig Reproductive Health Research Center (LE-REP), Leipzig University, Leipzig, Germany; 3 Department of Obstetrics, University of Leipzig Medical Center, Leipzig, Germany

**Keywords:** endothelial dysfunction, early-onset preeclampsia, late-onset preeclampsia, preeclampsia, platelet activation, thrombo-inflammation

## Abstract

**Background:**

Preeclampsia is a vascular complication of pregnancy with limited therapeutic options. It is associated with hypertension and an increase in angiogenic factor soluble fms-like tyrosine kinase-1 (sFlt-1)/placental growth factor. Based on its onset, preclampsia can be categorized into early-onset (E-PE) or late-onset (L-PE) preeclampsia. Thrombo-inflammation, hallmarked by maternal platelet activation and sterile inflammation, is associated with pathophysiology of preeclampsia. However, whether these mechanisms are differentially regulated in E-PE vs L-PE remains unknown.

**Objectives:**

We aim to study the role of maternal platelet activation, inflammation and endothelial dysfunction in E-PE vs L-PE.

**Methods:**

Flow-cytometry analysis of platelet activation (P-selectin and active αIIbβ3) was conducted in whole blood from pregnant women with E-PE, L-PE and gestational age-matched patients. Plasma was evaluated for interleukin (IL)-1β and soluble vascular cell adhesion molecule 1 (sVCAM-1).

**Results:**

An increase in P-selectin and active αIIbβ3 expressing platelets in both forms of preeclampsia (*n* = 22) was observed compared with their gestational age-matched controls (*n* = 18). Similarly, an increase in plasma IL-1β and sVCAM-1 was observed in both forms of preeclampsia, suggesting inflammation and endothelial dysfunction, respectively. Maternal platelet activation (P-selectin positive platelets) was linked with disease severity (sFlt-1/placental growth factor) and maternal plasma IL-1β and sVCAM-1 only in late-onset preeclampsia. A statistically significant correlation with αIIbβ3 expressing platelets and sFlt-1, IL-1β, and sVCAM-1 was not observed.

**Conclusions:**

These findings identify that thrombo-inflammation is regulated in L-PE and E-PE through likely disjunct mechanisms supporting a role of maternal factors (eg, maternal platelet activation) involved in L-PE. Further studies with a larger cohort of patients are required to fully elucidate the mechanistic relevance of these findings.

## Essentials

• Thrombo-inflammation in early-onset (E-PE) and late-onset preeclampsia (L-PE) is not well-studied.
• Human patients with L-PE and E-PE and gestational age-matched controls were studied.
• Platelet activation, inflammation, and endothelial dysfunction were increased in L-PE and E-PE.
• Thrombo-inflammation correlated with disease severity (soluble fms-like tyrosine kinase-1/placental growth factor) only in L-PE.

## 1 Introduction

Preeclampsia (PE) is a thrombo-inflammatory multifactorial human pregnancy syndrome affecting 5% to 7% pregnancies worldwide with limited therapeutic options and mechanistic insights [1]. It is characterized by de novo onset of hypertension after 20 weeks of gestation with subtypes of early (onset before 34 weeks) and late (onset after 34 weeks) diagnosis [2,3]. Early-onset PE (E-PE) is commonly associated with abnormal Doppler, intrauterine fetal growth restriction, and adverse maternal and neonatal outcomes [4,5]. In contrast, late-onset PE (L-PE) is mostly associated with normal or slight increased uterine resistance index, a low rate of fetal involvement, and more favorable perinatal outcomes [6,7]. There is clear placental pathology with E-PE, whereas in L-PE maternal factors are suggested to cause the late-onset disease. Although both forms of PE have placental dysfunction, the causes of the placental malperfusion and its timing differ. Both forms lead to secondary syncytiotrophoblast stress and release of proinflammatory factors into the maternal circulation [8,9]. Maternal factors may increase the risk on many levels for the two stages of PE and contribute to the risk for both early-onset and late-onset forms.

Platelet activation and associated sterile inflammation resulting in production of inflammatory cytokines such as interleukin [IL]-1β and IL-18, are known to be associated with pathophysiology of PE [10]. Furthermore, the release of placental antiangiogenic factors, such as soluble fms-like tyrosine kinase-1 (sFlt-1) and soluble endoglin promotes endothelial dysfunction in women with PE [11]. The increase in these antiangiogenic factors is responsible for the clinical manifestation of the disease and its severity, and reduction in these factors helps in restoring the angiogenic balance and possibly improving feto-maternal outcomes [[12], [13], [14]]. Increased maternal platelet activation in whole blood has been shown in L-PE patients [15]. Whether maternal platelet activation, the associated thrombo-inflammation, and endothelial dysfunction are differentially regulated in E-PE and L-PE and associated with disease severity, remain to be shown. This will provide us a rationale to use maternal platelet activation as a potential biomarker for the diagnosis of PE and to distinguish E-PE from L-PE at an early stage during pregnancy. Accordingly, this will allow us to better stratify the patients for efficient therapy and clinical management of patients with PE.

## 2 Materials and Methods

### 2.1 Human samples

**Figure 1:**
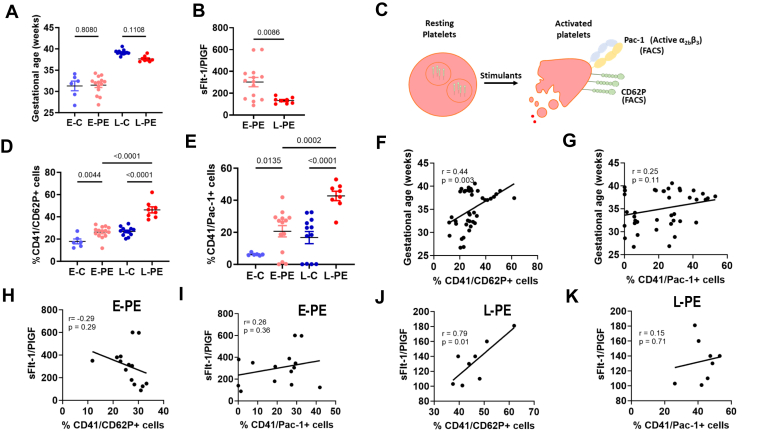
Platelet activation in early-onset and late-onset preeclampsia. (A) Bar graphs showing similar gestational age between early controls (E-C, *n* = 6) and early-onset preeclampsia (E-PE, *n* = 14) and between late controls (L-C, *n* = 12) and late-onset preeclampsia (L-PE, *n* = 8). (B) sFlt-1/PlGF ratio (bar graphs summarizing results) showing higher values in E-PE compared with patients with L-PE. (C) Schematic representation of different receptors on platelets showing that surface expression of CD62P and active αIIbβ3 integrin (Pac-1) is increased in activated platelets. (D–E) Bar graphs summarizing results from flow cytometry analysis showing an increased percentage of CD62P+ (D) and Pac-1+ (E) platelets (labeled using CD41+), suggesting increased platelet activation in both forms of preeclampsia compared with gestational age-matched controls (E-PE vs E-C and L-PE vs L-C). Activated platelets are higher in L-PE compared with E-PE. (F–G) Dot plots showing that platelet activation (F, CD62P+, but not G, Pac-1+) is positively correlated with gestational age in controls and patients with preeclampsia. (H–I) Correlation analysis between % activated platelets (H, CD62P+ and I, Pac-1+) and disease severity (sFlt-1/PlGF ratio) in patients with E-PE suggests no correlation with either platelet activation markers. (J–K) Correlation analysis between % activated platelets (J, CD62P+ and K, Pac-1+) and disease severity (sFlt-1/PlGF ratio) in patients with L-PE suggesting positive correlation with CD62P+ platelets. A–B, D–K: *n* = 6 (E–C), 14 (E-PE), 12 (L-C), 8 (L-PE); *P*-values are indicated in the graphs (A–B, D–E: anova, F–K, Pearson’s correlation). *P* < .05 was considered statistically significant. PlGF, placental growth factor; sFlt-1, soluble fms-like tyrosine kinase-1.

Human blood samples from singleton pregnancies complicated with preeclampsia (*n* = 22) and gestational age-matched normotensive controls (*n* = 18) were collected at the University Hospital Leipzig in accordance with the guidelines and with the approval of the local ethics committee (Figures 1A and B). Preeclampsia was diagnosed in accordance with published guidelines by the International Society for the Study of Hypertension in Pregnancy (ISSHP) [16]. Preeclampsia was classified into early-onset (<34+0 gestational weeks, *n* = 14)) and late-onset (≥34+0 gestational weeks, *n* = 8), based on gestational age at diagnosis. Sodium citrate anticoagulated blood was collected from the patients and processed within 30 minutes (separation of plasma or separation and storage of plasma at −80 °C) for analysis. Women receiving aspirin or heparin and those having known conditions affecting blood coagulation were excluded from this study.

### 2.2 Platelet activation studies

Platelet activation was evaluated with flow cytometry using CD41a-eFlour450 (Invitrogen, integrin αIIb, used for platelet identification), CD62P-APC (Biolegend, P-selectin, used for identification of activated platelets), and Pac-1-FITC (BD Biosciences, activated conformation of αIIbβ3, used for identification of activated platelets) fluorescently labeled antibodies. Whole citrate blood was diluted in cell wash buffer (BD). One part was stained with fluorescently labeled antibodies for 20 minutes. The other was unstained (negative control). The adenosine diphosphate activated platelets were used as a positive control. All groups were fixed with 2% paraformaldehyde followed by washing with cell wash buffer. Acquisition was performed on Attune flow cytometer (Invitrogen).

### 2.3 Enzyme-linked immunosorbent assay

Platelet poor plasma was separated from whole blood by centrifugation for 100 g at 10 minutes followed by a second centrifugation at 1500 g for 10 minutes and used for enzyme-linked immunosorbent assay (ELISA) measurements for IL-1β and soluble vascular cell adhesion molecule 1 (sVCAM-1) (R&D biosystems). ELISA was conducted according to manufacturer’s protocol for human IL-1β and human sVCAM-1 ELISAs.

### 2.4 Measurement of sFlt-1/placental growth factor

The levels of sFlt-1 and placental growth factor (PlGF) were quantified using Elecsys electrochemiluminescent immunoassays on a Roche Cobas 8000 clinical chemistry analyzer. These measurements were conducted in accordance with International Organization for Standardization 15180 and International Organization for Standardization 17025 accreditation standards.

### 2.5 Statistical analysis

Data are summarized as the means ± SEMs. Statistical analyses were performed with Student’s *t*-test or anova, as appropriate. Post hoc comparisons of anova were corrected with Šídák’s multiple comparisons test. The Kolmogorov–Smirnov (KS) test or D’Agostino–Pearson normality-test was used to determine whether the data are consistent with a Gaussian distribution. Pearson’s correlation coefficient was calculated based on simple linear regression. Statistical analyses performed are delineated in each figure legend using GraphPad Prism. Statistical significance was accepted at *P*-values of < .05.

## 3 Results and Discussion

### 3.1 Increased platelet activation in early and late-onset PE

We first evaluated platelet activation in patients diagnosed with early-onset preeclampsia (E-PE), late-onset preeclampsia (L-PE) and their respective gestational age-matched controls (Figures 1A and B). We conducted flow cytometry analysis of whole blood and studied surface expression of platelet activation markers P-selectin (CD62P) and activation-induced conformational epitope of αIIbβ3 integrin (Pac-1; CD41/CD61) in CD41a+ platelets at baseline without an additional platelet activation agonist (Figure 1C). We observed an increased prevalence of P-selectin (CD62P)-positive platelets in both PE groups compared with respective gestational age-matched controls suggesting increased platelet activation in both groups (Figure 1D). Similarly, E-PE and L-PE showed increased active αIIbβ3 integrin (Pac-1)-positive platelets compared with gestational age-matched controls (Figure 1E). Moreover, L-PE patients showed a significantly higher percentage of activated platelet population compared with E-PE based on both markers (Figures 1D and E). These findings suggested that women with L-PE have higher platelet activation than women with E-PE, but also that both forms are affected by increased activation compared with unaffected pregnancies.

The L-PE differs from E-PE with regard to time of disease onset during gestation. Moreover, L-PE is thought to be largely of maternal origin, whereas E-PE is considered to be placental in origin. %CD62P+ platelets positively correlated with gestational age when the data was combined from all groups. This suggests that with increase in gestational age, there is an increase in activated platelets (Figure 1F). However, no correlation was observed between gestational age and Pac-1+ platelets (Figure 1G). The L-PE had higher platelet activation compared with E-PE. However, baseline platelet activation, eg, platelet activation in women without PE, was likewise increased at the later pregnancy stage.

### 3.2 Platelet activation correlates with sFlt-1/PlGF in late-onset PE

Plasma sFlt-1/PlGF ratio is a biomarker of preeclampsia and is suggestive of disease severity. Previous studies suggest that patients with E-PE generally have higher plasma sFlt-1/PlGF compared with L-PE indicating the placental origin of E-PE [17]. We compared plasma sFlt-1/PlGF in these patients and whether they correlated with markers of platelet activation, inflammation, and endothelial dysfunction in these patients. In our cohort, patients with E-PE had higher sFlt-1/PlGF ratio compared with L-PE (Figure 1B). Interestingly, sub-group analysis suggested that sFlt-1/PlGF correlated with platelet activation marker (CD62P) only in L-PE and not in patients with E-PE (Figures 1H and J). However, a correlation of sFlt-1/PlGF with Pac-1 was not observed (Figure 1I and K). This suggested that although patients with E-PE have higher sFlt-1, CD62P+ activated platelets correlate with disease severity only in L-PE.

### 3.3 Increased thrombo-inflammation in L-PE is associated with sFlt-1/PlGF

**Figure 2:**
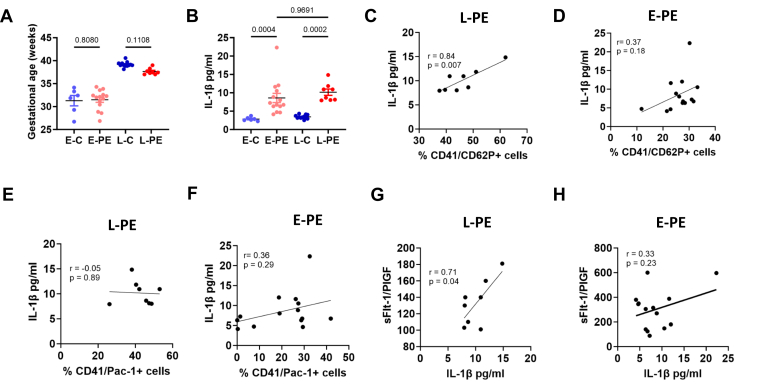
Increased interleukin [IL]-1β in early-onset and late-onset preeclampsia. (A) Bar graphs showing similar gestational age between early controls (E-C, *n* = 6) and early-onset preeclampsia (E-PE, *n* = 14) and between late controls (L-C, *n* = 12) and late-onset preeclampsia (L-PE, *n* = 8). (B) Bar graphs showing an increased plasma IL-1β in both forms of preeclampsia compared with gestational age-matched controls (E-PE vs E-C and L-PE vs L-C), suggesting increased inflammation. (C–F) Correlation analysis between % activated platelets (C, D, CD62P+ and E, F, Pac-1+) and inflammation (IL-1β levels) suggesting positive correlation with CD62P+ platelet activation marker in L-PE (C) and no correlation in E-PE (D,F). (G–H) Correlation analysis between disease severity (sFlt-1/PlGF ratio) and inflammation (IL-1β levels) suggesting a positive correlation in L-PE (G) and no correlation in E-PE (H). A–H: *n* = 6 (E-C), 14 (E-PE), 12 (L-C), 8 (L-PE); *P*-values are indicated in the graphs (A, B: anova, C–H: Pearson’s correlation). *P* < .05 was considered statistically significant. PlGF, placental growth factor; sFlt-1, soluble fms-like tyrosine kinase-1.

Platelet activation is associated with sterile inflammation and increase inflammatory cytokines (eg, IL-1β and IL-6) in PE, thereby resulting in thrombo-inflammation [18,19]. Increased plasma levels of IL-1β were observed in both E-PE and L-PE compared with respective gestational age-matched controls (Figures 2A and B). There was no significant difference between E-PE and L-PE. However, a positive correlation of platelet activation marker (CD62P) with plasma IL-1β levels was observed only in patients with L-PE (Figure 2C). Increased IL-1β levels in patients with E-PE did not show any statistically significant correlation with platelet activation markers (Figure 2D). Again, no correlation was observed based with Pac-1+ platelets (Figures 2E and F). The increased IL-1β correlated with plasma sFlt-1 only in L-PE patients and showed no correlation in patients with E-PE (Figures 2G and H). These data suggest that while both groups have inflammation, a higher platelet activation in L-PE may contribute to increased thrombo-inflammation and disease severity. On the contrary, inflammation in E-PE may be due to yet unidentified different reasons (eg, altered immune-cell landscape).

### 3.4 Increased endothelial dysfunction in early and late-onset PE

**Figure 3:**
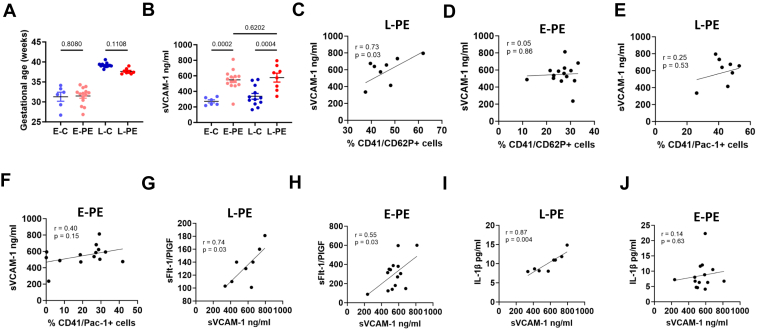
Increased sVCAM-1 in early-onset and late-onset preeclampsia. (A) Bar graphs showing similar gestational age between early controls (E-C, *n* = 6) and early-onset preeclampsia (E-PE, *n* = 14) and between late controls (L-C, *n* = 12) and late-onset preeclampsia (L-PE, *n* = 8). (B) Bar graphs showing an increased plasma sVCAM-1 in both forms of preeclampsia compared with gestational age-matched controls (E-PE vs E-C and L-PE vs L-C) suggesting endothelial dysfunction. (C–F) Correlation analysis between % activated platelets (C, D, CD62P+ and E, F, Pac-1+) and endothelial dysfunction (sVCAM-1 levels) suggesting positive correlation with CD62P+ platelets in L-PE (C) and no correlation in E-PE (D,F). (G–H) Correlation analysis between endothelial dysfunction (sVCAM-1 levels) and disease severity (sFlt-1/PlGF ratio) suggesting a positive correlation in both L-PE (G) and E-PE (H). (I–J) Correlation analysis between endothelial dysfunction (sVCAM-1 levels) and inflammation (IL-1β levels) suggesting a positive correlation between endothelial dysfunction (sVCAM-1) and inflammation (IL-1β) in L-PE (I) but no correlation in E-PE (J). A–J: *n* = 6 (E-C), 14 (E-PE), 12 (L-C), 8 (L-PE); *P*-values are indicated in the graphs (A, B: anova, C–J: Pearson’s correlation). *P* < .05 was considered statistically significant. IL, interleukin; PlGF, placental growth factor; sFlt-1, soluble fms-like tyrosine kinase-1; sVCAM1, soluble vascular cell adhesion molecule-1.

Endothelial dysfunction, platelet activation, and thrombo-inflammation are mechanistically interlined in PE. Therefore, we next evaluated plasma levels of sVCAM-1 as a marker of endothelial dysfunction in patients with E-PE and L-PE. Both E-PE and L-PE showed increased plasma levels of sVCAM-1 compared with respective gestational age-matched controls (Figures 3A and B). There was no significant difference in either of the markers between E-PE and L-PE, suggesting endothelial dysfunction in both groups. Endothelial dysfunction (increased plasma sVCAM-1) correlated with platelet activation (CD62P, but not Pac-1), sFlt-1/PlGF, and IL-1β in L-PE (Figures 3C, E, G, and I). In patients with E-PE, soluble vascular cell adhesion molecule-1 was associated with sFlt-1, but not with IL-1β or platelets (Figures 3D, F, H, and J). Taken together, these data suggest that endothelial dysfunction is linked to disease severity in E-PE and L-PE. However, endothelial dysfunction appears to be linked with platelet activation and thrombo-inflammation in L-PE, suggesting a mechanistic interaction of endothelial dysfunction and platelet activation in L-PE. On the contrary, endothelial dysfunction appears to be independent of platelet activation in E-PE, suggesting a different pathomechanism.

**Figure 4:**
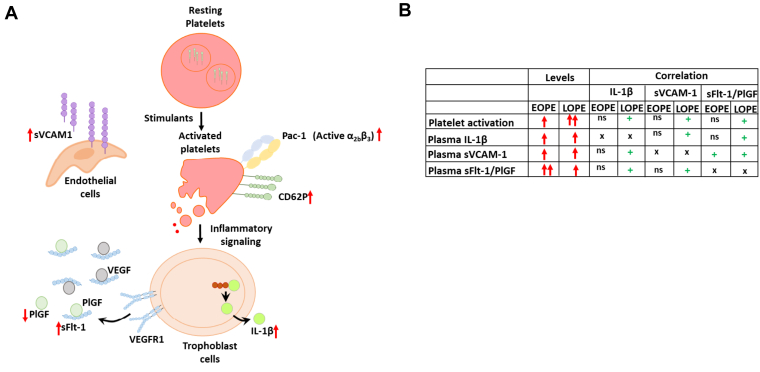
Mechanism of thrombo-inflammation in early-onset and late-onset preeclampsia. (A) Schematic representation of potential mechanism by which platelet activation promotes inflammatory signaling resulting in placental thrombo-inflammation. This is accompanied by increase in plasma sFlt-1/PlGF suggesting disease severity and increase in soluble vascular cell adhesion molecule-1 (sVCAM-1) suggesting endothelial dysfunction in women with preeclampsia. The mechanisms by which this is differentially regulated in E-PE and L-PE remains unclear. (B) Schematic representation summarizing the results of the study. Upward red-arrow indicate increased levels, + sign indicates a positive correlation, ns indicates a nonsignificant correlation, and x indicates correlation not relevant. IL, interleukin; PlGF, placental growth factor; sFlt-1, soluble fms-like tyrosine kinase-1.

The role of platelet activation, thrombo-inflammation, and endothelial dysfunction in early-onset preeclampsia and late-onset preeclampsia is still debated. We here show that although these pathomechanisms are involved in both E-PE and L-PE, they appear to be linked with each other and disease severity (sFlt-1/PlGF) only in L-PE. Specifically, platelet activation is associated with increased inflammation (IL-1β), antiangiogenic factors (sFlt-1), and endothelial dysfunction (sVCAM-1) in L-PE (Figure 4). This supports the theory that L-PE manifests due to maternal factors (eg, maternal platelets) affecting the placental function, resulting in increase in sFlt-1 and PE symptoms [20]. On the contrary, the increase in platelet activation, inflammation, and endothelial dysfunction in E-PE may be associated with a complex disease etiology in E-PE. While a direct association of E-PE with its placental origin cannot be concluded based on our results, the increased platelet activation in E-PE is less likely to be the disease driver. We speculate that platelet activation and thrombo-inflammation in E-PE may contribute to worsening of the disease as a consequence of placental and endothelial dysfunction.

A positive correlation between gestational age and platelet activation (CD41/CD62P+ cells) was observed suggesting that the gestational duration impacts platelet activation. This suggests that studying platelet activation mechanisms during gestation may provide important insights into potentially differential mechanisms of thrombo-inflammation in E-PE and L-PE.

Low-dose aspirin is recommended during pregnancy to reduce the risk of PE. Several clinical studies suggest its beneficial use and reduced chances of preeclampsia and associated complications in women with high risk [21]. Aspirin causes irreversible inhibition of cyclooxygenase (COX), which suppresses the production of prostaglandins and thromboxane thereby preventing platelet activation [22]. The COX signaling and production of arachidonic acid metabolites are essential in mediating several biological functions, including immune-response, blood pressure, and vasodilation [23]. Therefore, aspirin can effectively blunt a variety of proinflammatory factors responsible beyond inhibiting platelet activation [22,24]. Furthermore, noncanonical (eg, non-COX) mediated effects of aspirin (eg, acetylation) have been proposed but are not well understood in the context of PE [22,25]. Of note, a reduced preeclampsia risk by aspirin has been suggested largely in E-PE [26]. In contrast, we did not see a correlation between increased platelet activation and sFlt-1/PlGF in E-PE. This suggest that the protection conveyed by aspirin in E-PE may be attributed to its other biological and antiinflammatory effects.

In the context of L-PE, where we observed an association of thrombo-inflammation with sFlt-1/PlGF, monitoring platelet activation and thrombo-inflammation may be helpful for early diagnosis of L-PE. Furthermore, it may be interesting to explore the beneficial effects of other antiplatelet drugs. A beneficial effect of other antiplatelet agents, such as ticagrelor and clopridogel in pregnancy and PE have been indicated [27]. Systematic preclinical and clinical studies evaluating the safety and efficacy of these drugs to prevent L-PE are warranted. Moreover, drugs that have dual actions in preventing both platelet activation and inflammation or combination therapies may provide a benefit in delaying the delivery in L-PE.

A major limitation of the study is the small sample size. Moreover, the parameters did not show correlation with Pac-1, likely due to heterogeneous data using this marker and low sample size. Therefore, the results obtained within the study should be validated with a larger, preferably multicentric cohort. Furthermore, additional markers for inflammation and endothelial dysfunction should be included to draw reliable conclusions on the effect of thrombo-inflammation. Specifically, we could not establish a causality between platelet activation and endothelial dysfunction in L-PE. This is often a limitation observed in clinical studies, where targeted interventions are difficult to conduct. However, it should be noted that this cohort is composed of women without any other medical conditions and with otherwise uneventful singleton pregnancies. Consequently, we think it to be unlikely that the results of these analyses are caused by mechanisms other than preeclampsia.

In summary, platelet activation could be observed in both E-PE and L-PE, with higher overall levels of activation in the latter form of PE. In addition, increasing platelets activation was associated with higher levels of cytokines (ie, IL-1β levels) in patients with L-PE. Further studies are needed to establish the role of thrombo-inflammation in the pathophysiology of PE.

## References

[1] Lisonkova S., Joseph K.S. (2013). Incidence of preeclampsia: risk factors and outcomes associated with early- versus late-onset disease. Am J Obstet Gynecol.

[2] Hypertension in pregnancy (2013). Report of the American College of Obstetricians and Gynecologists’ Task Force on Hypertension in Pregnancy. Obstet Gynecol.

[3] Wójtowicz A., Zembala-Szczerba M., Babczyk D., Kołodziejczyk-Pietruszka M., Lewaczyńska O., Huras H. (2019). Early- and Late-Onset Preeclampsia: A Comprehensive Cohort Study of Laboratory and Clinical Findings according to the New ISHHP Criteria. Int J Hypertens.

[4] Tranquilli A.L., Brown M.A., Zeeman G.G., Dekker G., Sibai B.M. (2013). The definition of severe and early-onset preeclampsia. Statements from the International Society for the Study of Hypertension in Pregnancy (ISSHP). Pregnancy Hypertens.

[5] van Esch J.J.A., van Heijst A.F., de Haan A.F.J., van der Heijden O.W.H. (2017). Early-onset preeclampsia is associated with perinatal mortality and severe neonatal morbidity. J Matern Fetal Neonatal Med.

[6] Valensise H., Vasapollo B., Gagliardi G., Novelli G.P. (2008). Early and late preeclampsia: two different maternal hemodynamic states in the latent phase of the disease. Hypertension.

[7] Llurba E., Carreras E., Gratacós E., Juan M., Astor J., Vives A. (2009). Maternal history and uterine artery Doppler in the assessment of risk for development of early- and late-onset preeclampsia and intrauterine growth restriction. Obstet Gynecol Int.

[8] Visser N., van Rijn B.B., Rijkers G.T., Franx A., Bruinse H.W. (2007). Inflammatory changes in preeclampsia: current understanding of the maternal innate and adaptive immune response. Obstet Gynecol Surv.

[9] Aly A.S., Khandelwal M., Zhao J., Mehmet A.H., Sammel M.D., Parry S. (2004). Neutrophils are stimulated by syncytiotrophoblast microvillous membranes to generate superoxide radicals in women with preeclampsia. Am J Obstet Gynecol.

[10] Guan X., Fu Y., Liu Y., Cui M., Zhang C., Zhang Q. (2023). The role of inflammatory biomarkers in the development and progression of pre-eclampsia: a systematic review and meta-analysis. Front Immunol.

[11] Agarwal I., Karumanchi S.A. (2011). Preeclampsia and the anti-angiogenic state. Pregnancy Hypertens.

[12] Maynard S.E., Karumanchi S.A. (2011). Angiogenic factors and preeclampsia. Semin Nephrol.

[13] Cerdeira A.S., Vatish M., Lecarpentier E. (2020). One step closer to a cure for preeclampsia?: boosting the natural affinity of VEGF (vascular endothelial growth factor) to sFlt (soluble fms-like tyrosine kinase)-1. Hypertension.

[14] Thadhani R., Hagmann H., Schaarschmidt W., Roth B., Cingoez T., Karumanchi S.A. (2016). Removal of soluble Fms-like tyrosine Kinase-1 by dextran sulfate apheresis in preeclampsia. J Am Soc Nephrol.

[15] Holthe M.R., Staff A.C., Berge L.N., Lyberg T. (2004). Different levels of platelet activation in preeclamptic, normotensive pregnant, and nonpregnant women. Am J Obstet Gynecol.

[16] Brown M.A., Lindheimer M.D., de Swiet M., Van Assche A., Moutquin J.M. (2001). The classification and diagnosis of the hypertensive disorders of pregnancy: statement from the International Society for the Study of Hypertension in Pregnancy (ISSHP). Hypertens Pregnancy.

[17] Schaarschmidt W., Rana S., Stepan H. (2013). The course of angiogenic factors in early- vs late-onset preeclampsia and HELLP syndrome. J Perinat Med.

[18] Shirasuna K., Karasawa T., Takahashi M. (2020). Role of the NLRP3 inflammasome in preeclampsia. Front Endocrinol (Lausanne).

[19] Kohli S., Ranjan S., Hoffmann J., Kashif M., Daniel E.A., Al-Dabet M.M. (2016). Maternal extracellular vesicles and platelets promote preeclampsia via inflammasome activation in trophoblasts. Blood.

[20] Staff A.C. (2019). The two-stage placental model of preeclampsia: an update. J Reprod Immunol.

[21] Wang Y., Guo X., Obore N., Ding H., Wu C., Yu H. (2022). Aspirin for the prevention of preeclampsia: a systematic review and meta-analysis of randomized controlled studies. Front Cardiovasc Med.

[22] Ornelas A., Zacharias-Millward N., Menter D.G., Davis J.S., Lichtenberger L., Hawke D. (2017). Beyond COX-1: the effects of aspirin on platelet biology and potential mechanisms of chemoprevention. Cancer Metastasis Rev.

[23] Ricciotti E., FitzGerald G.A. (2011). Prostaglandins and inflammation. Arterioscler Thromb Vasc Biol.

[24] Smith W.L., DeWitt D.L., Garavito R.M. (2000). Cyclooxygenases: structural, cellular, and molecular biology. Annu Rev Biochem.

[25] Flower R. (2003). What are all the things that aspirin does?. BMJ.

[26] Rolnik D.L., Wright D., Poon L.C., O’Gorman N., Syngelaki A., de Paco Matallana C. (2017). Aspirin versus Placebo in Pregnancies at High Risk for preterm Preeclampsia. N Engl J Med.

[27] Verbruggen M., Mannaerts D., Muys J., Jacquemyn Y. (2015). Use of ticagrelor in human pregnancy, the first experience. BMJ Case Rep.

